# Supplementary material for: Fast and covariate-adaptive method amplifies detection power in large-scale multiple hypothesis testing
Source: Nat Commun. 2019 Jul 31;10:3433. doi: 10.1038/s41467-019-11247-0 (PMC6668431; doi:10.1038/s41467-019-11247-0)
Supplement: Supplementary file 1 — Supplementary Information [file 41467_2019_11247_MOESM1_ESM.pdf]

Fast and covariate-adaptive method amplifies detection power in large-scale multiple testing

Zhang et al.

Supplementary Information

Supplementary Figures

a number of selected eQTLs in the two colon tissues

unit: million	BH	SBH	IHW	AdaFDR	AdaFDR (aug)	AdaFDR (ctrl)
Colon_ Sigmoid	0.72	0.73 (+1.0%)	0.77 (+6.6%)	0.97 (+34.6%)	1.18 (+63.9%)	1.03 (+43.0%)
Colon_ Transverse	0.87	0.88 (+0.9%)	0.92 (+5.5%)	1.14 (+31.8%)	1.33 (+52.9%)	1.16 (+33.3%)

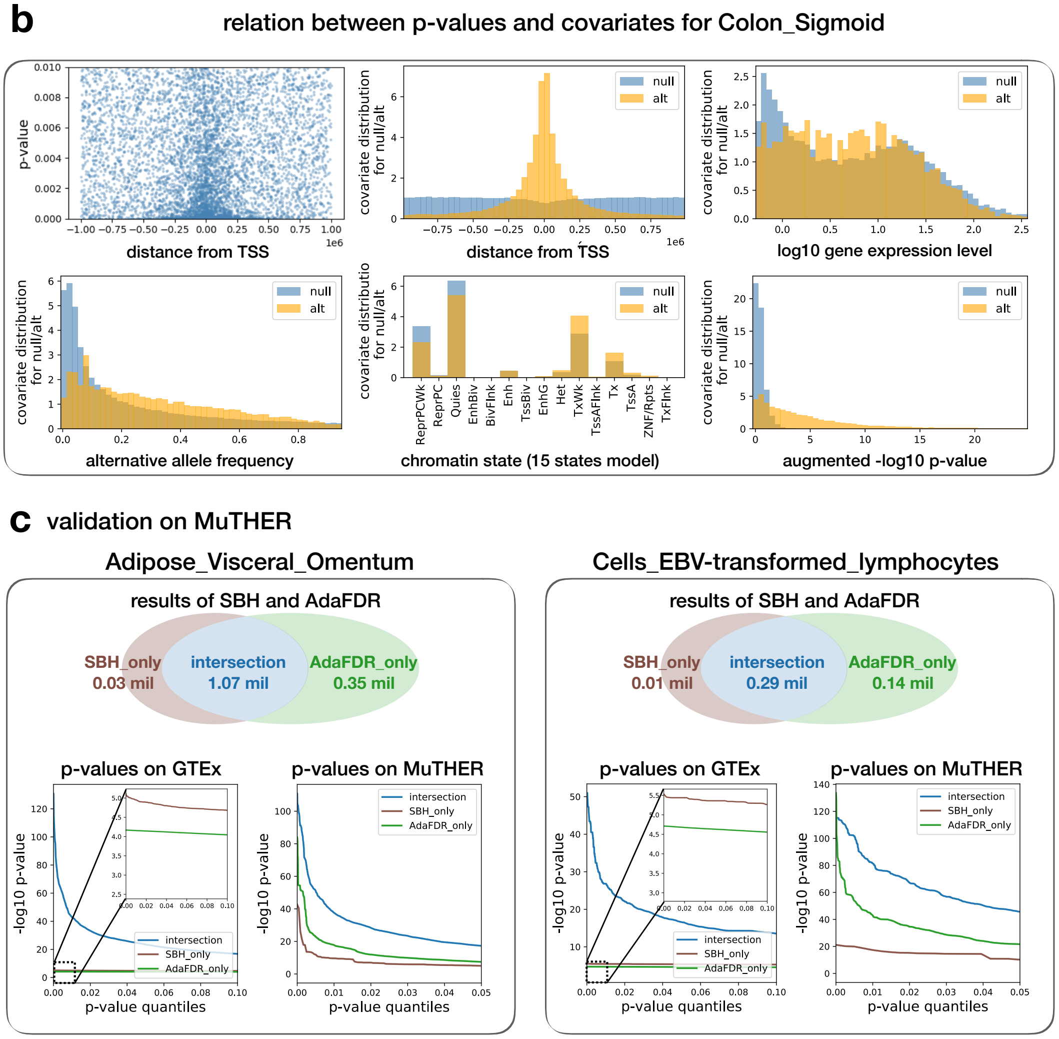

Supplementary Figure 1. Additional results on the GTEx data. (a) Results on the two colon tissues. (b) Feature visualization for Colon_Sigmoid (c) Validation for GTEx Adipose_Visceral_Omentum using the MuTHER adipose eQTL data (left) and for GTEx Cells_EBV-transformed_lymphocytes using the MuTHER lymphocytes (LCL) eQTL data (right).

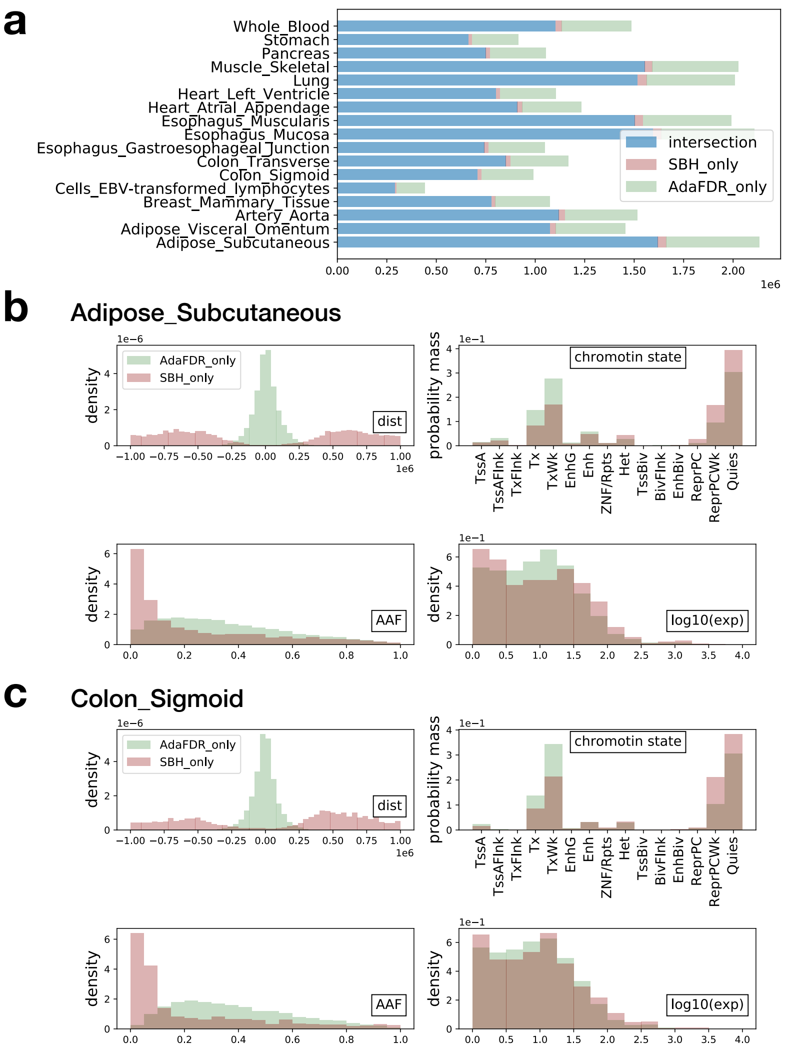

Supplementary Figure 2. Comparing the `AdaFDR` result and the SBH result on the GTEx data. (a) Result comparison between SBH and `AdaFDR`. For all 17 GTEx tissues, `AdaFDR` missed a tiny proportion of SBH discoveries while having substantially more other discoveries. Source data are provided as a Source Data file. (b-c) The marginal distribution of `AdaFDR`-only discoveries and SBH-only discoveries over each covariate is shown for the tissue Adipose_Subcutaneous and Colon_Sigmoid respectively. There is a higher proportion of `AdaFDR`-only discoveries at locations where 1) the distance from TSS is small (upper left); 2) the SNP has an active chromatin state (upper right); 3) the SNP AAF is close to 0.5 (lower left); 4) the gene expression level is neither too high or too low (lower right). All these match the enrichment pattern of eQTLs (Results), indicating that the `AdaFDR`-only discoveries are more biologically relevant.

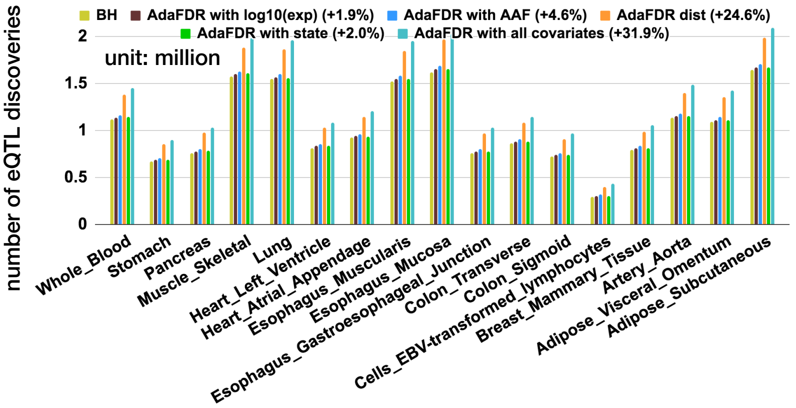

Supplementary Figure 3. Contribution of each covariate for the GTEx data. To investigate the individual contribution of different covariates, we run `AdaFDR` using each covariate separately for all tissues in the GTEx experiment. We use a nominal FDR level of 0.01 same as before. The distance from TSS is most informative while others have have smaller but still notable effects. Interestingly, the combined improvement of using all covariates (31.9%) is similar to the sum of the four individual improvements (33.0%), indicating that the four covariates carry very different information regarding the hypotheses. Source data are provided as a Source Data file.

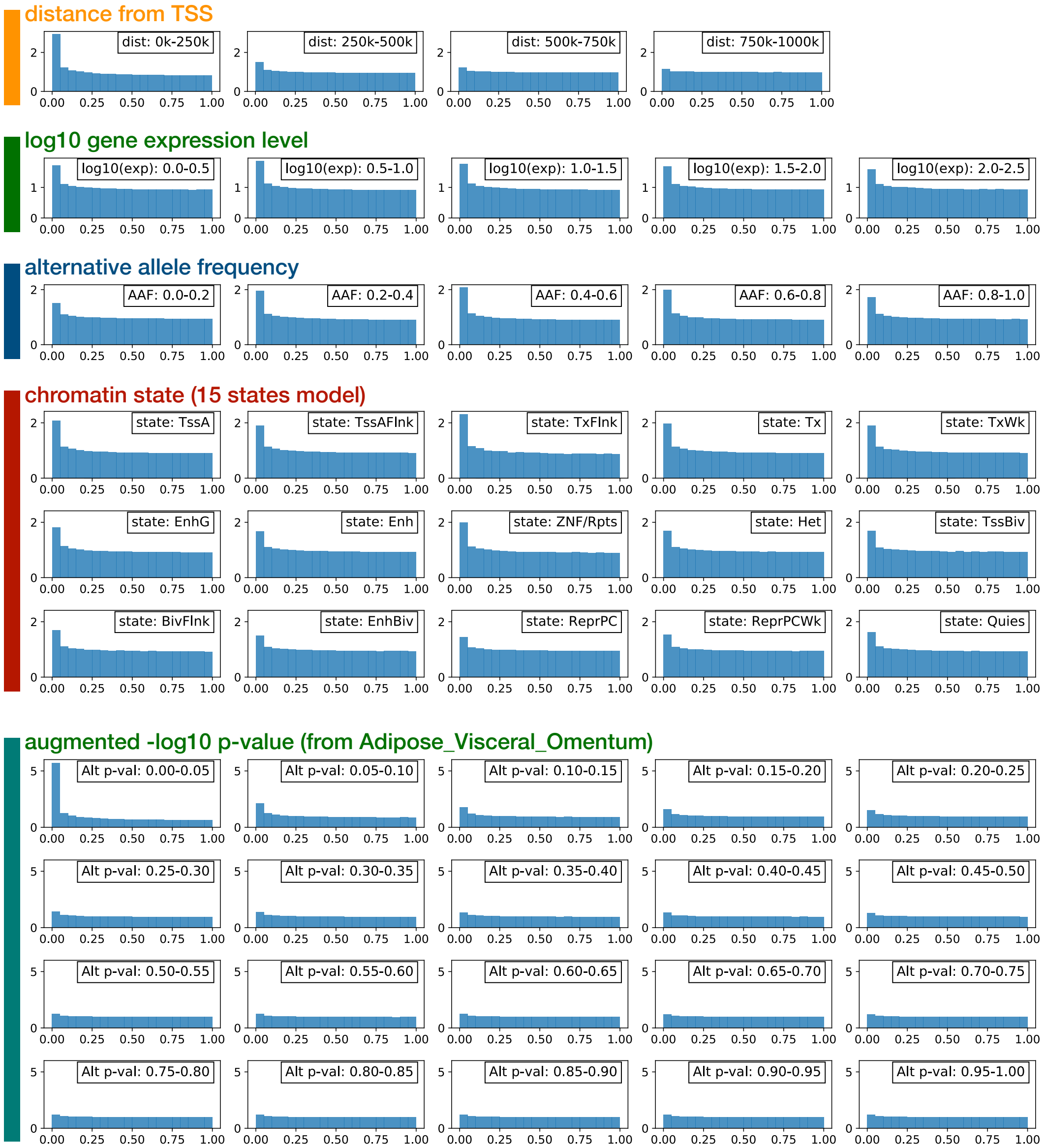

Supplementary Figure 4. Assumption check for the GTEx Adipose_Subcutaneous data. To verify the algorithm assumption (Theorem 1) for the GTEx experiments, we plot the p-value histograms stratified by each covariate separately for the tissue Adipose_Subcutaneous. All histograms show a mixture of a uniform distribution and an enrichment of small p-values to the left, indicating that the null p-values are uniformly distributed independent of the covariate.

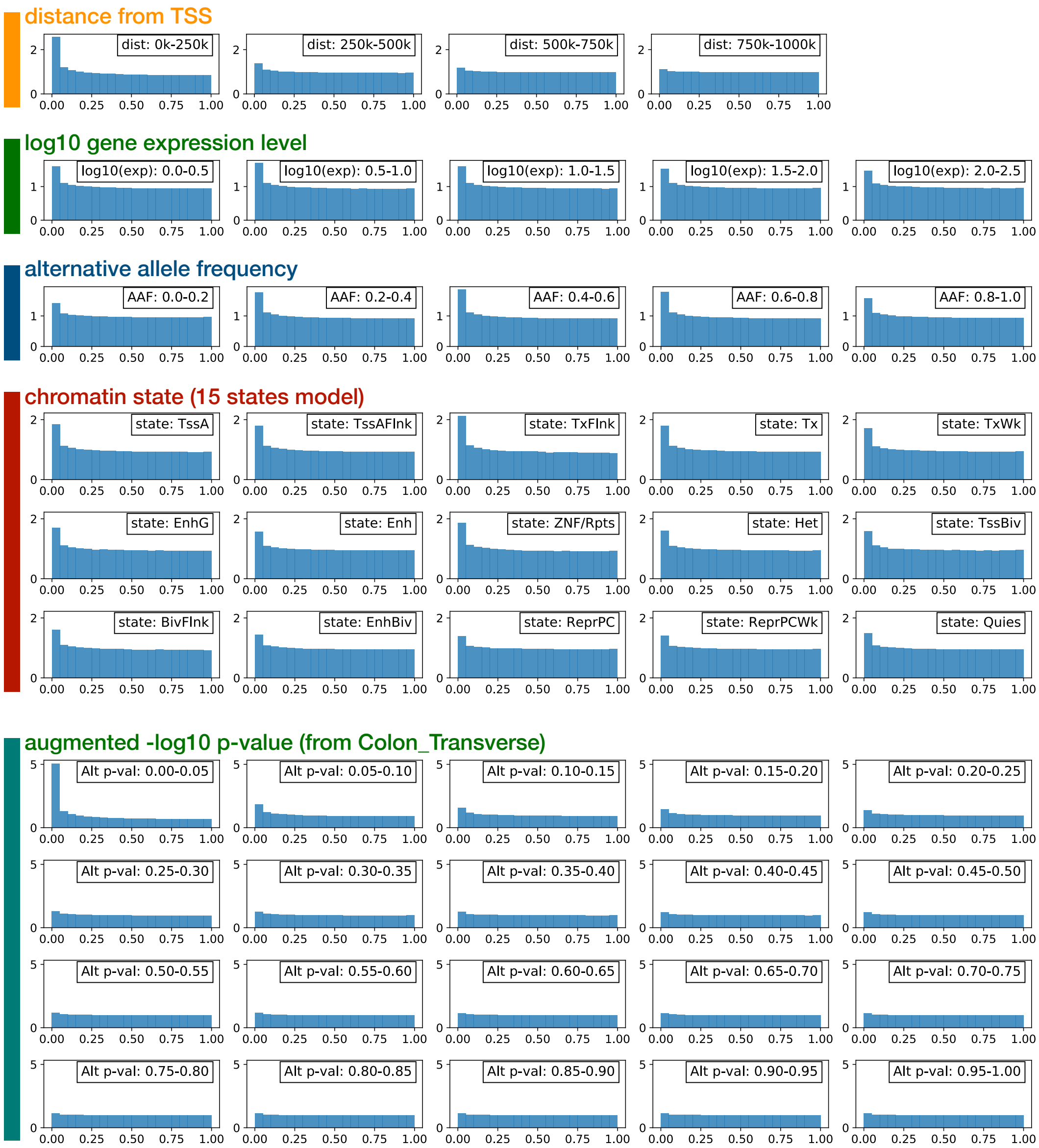

Supplementary Figure 5. Assumption check for the GTEx Colon_Sigmoid data. P-value histograms stratified by each covariate separately for the tissue Colon_Sigmoid. Similar to Supplementary Figure 4.

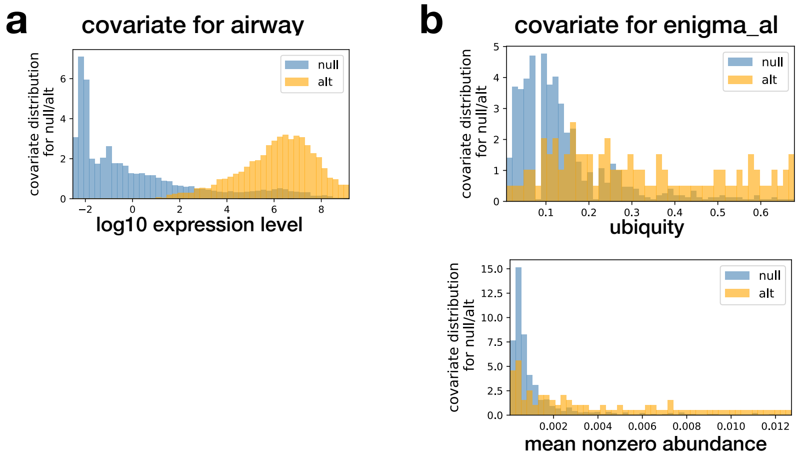

Supplementary Figure 6. Additional covariate visualizations. (a) The covariate visualization for the RNA-Seq airway data. (b) The covariate visualization for the microbiome enigma_al data. Top: ubiquity; bottom: mean nonzero abundance.

	discoveries (std)	reproduced discoveries %
**small_GTEx: Adipose_Subcutaneous**	1491 (41)	94.5%
**small_GTEx: Adipose_Visceral_Omentum**	1396 (96)	89.5%
**RNA-Seq: Bottomly**	2147 (38)	93.6%
**RNA-Seq: Pasilla**	830 (15)	94.4%
**RNA-Seq: airway**	6041 (33)	97.2%
**microbiome: enigma_ph**	119 (8)	87.8%
**microbiome: enigma_al**	480 (46)	82.4%
**proteomics**	408 (18)	89.6%
**fMRI: auditory**	1066 (10)	96.9%
**fMRI: imagination**	2233 (12)	97.6%

Supplementary Figure 7. Algorithm stability. `AdaFDR` may produce slightly different results in different runs on the same dataset due to its inherent randomness. To showcase its stability, we repeat all 10 experiments in Figure 3a 50 times with different random seeds. As shown in the first column of the table, the number of discoveries of the 50 repetitions are highly consistent. Furthermore, for each of the 50 repetitions, we run AdaFDR for a second time and report the proportion of reproduced discoveries in the second column of the table (number of overlapped discoveries in both runs divided by average number of discoveries in the first run). The average replication rate is 92.4% across the ten datasets, indicating good stability of the algorithm. The two microbiome datasets have relatively lower replication rate (87.8% and 82.4%, respectively), due to their smaller data size ($\sim$ 4000 hypotheses).

a more simulations for FDP and power

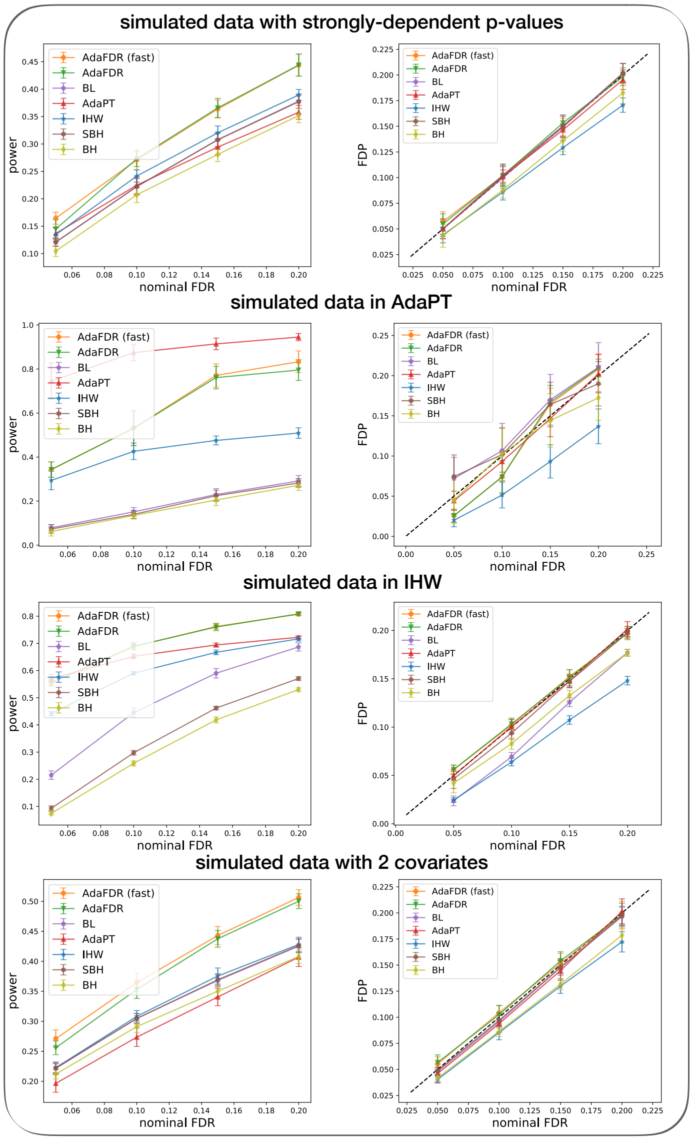

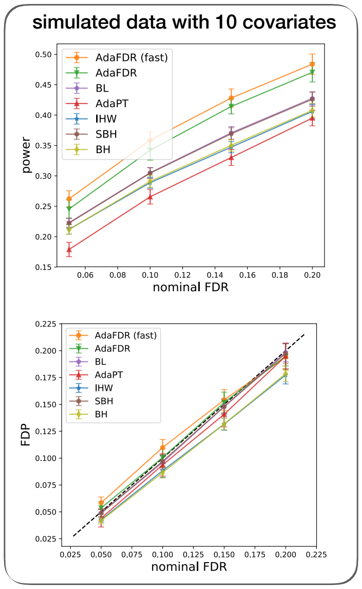

b comparison with NeuralFDR

	NeuralFDR	AdaFDR
**2DGM**	18844	**20182**
**5DGM**	18364	**19832**
**GTEx_ NeuralFDR**	**37195**	37141

Supplementary Figure 8. More simulation studies. 95% confidence intervals are provided for all panels. (a) Additional simulations for FDP and power. Descriptions of the data are in Supplementary Note 2.6. (b) Comparison between `NeuralFDR` and `AdaFDR`.

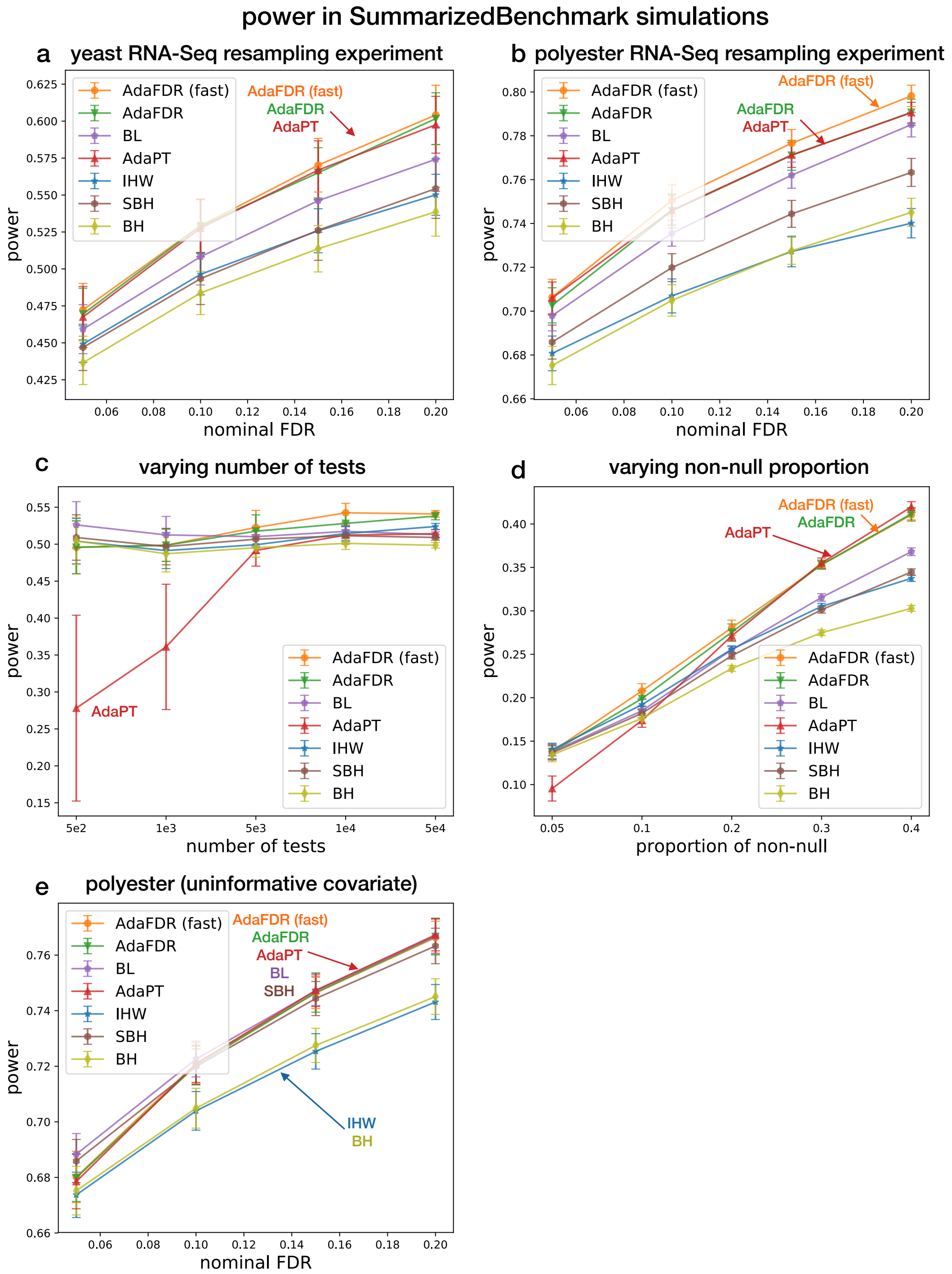

Supplementary Figure 9. SummarizedBenchmark simulations[11] (power). Power in five SummarizedBenchmark simulations[11] with the corresponding FDP shown in Supplementary Figure 10. Panels a-d correspond to Figure 3 in[11] while panel e corresponds to the first row of Table S2 in[11]. Ten resamplings were done for RNA-Seq experiments (a,b,e) while twenty were done for others; 95% confidence intervals are provided. Panels a, b are two RNA-Seq spike-in resampling experiments with an informative covariate, panel c contains a simulated data with the number of tests varying from 500 to 50k, while panel d contains a simulated data with the non-null proportion of tests varying from 0.95 to 0.6. In all four experiments, `AdaFDR` and AdaPT have the highest power (with `AdaFDR` being slightly better). We note that AdaPT does not have such high power in the same experiments in[11]. This is probably because we used adapt_gam while adapt_glm is used in[11]; the former has a better performance but takes a longer time to run. Panel e uses the same set of p-values as panel b but with an uninformative covariate. We can see the performance of IHW reduces to BH while others reduce to SBH, a phenomenon also mentioned in[11]. `AdaFDR` maintains high power here indicating that it does not overfit the uninformative covariate.

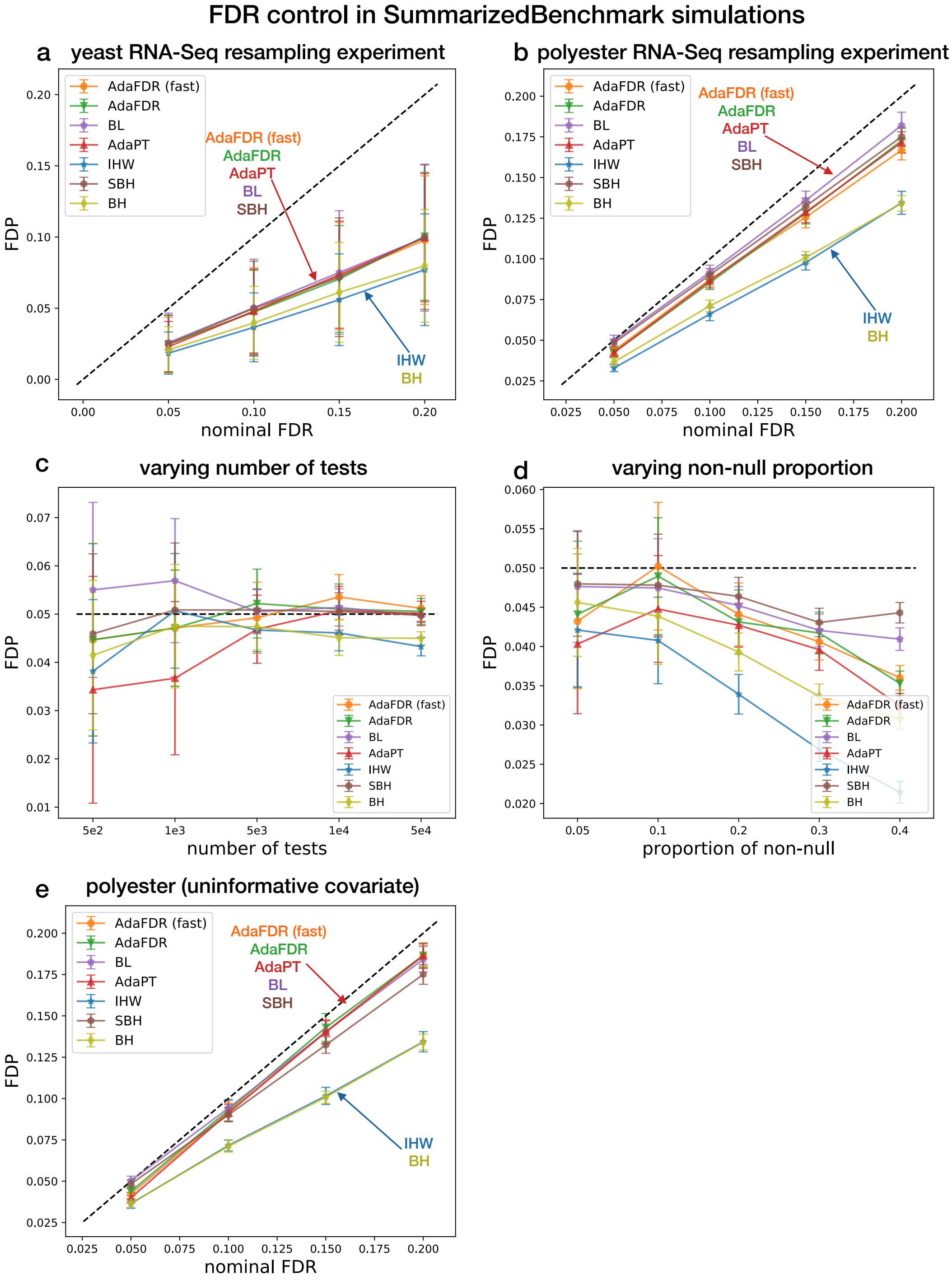

Supplementary Figure 10. SummarizedBenchmark simulations[11] (FDP). 95% confidence intervals are provided for all panels. FDR control in five SummarizedBenchmark simulations[11] with the corresponding power shown in Supplementary Figure 9. The detailed description of the data can also be found in Supplementary Figure 9. All methods control the FDR accurately, except in panel c, where BL slightly exceeds the nominal FDR level when the number of tests is small.

Supplementary Notes

1 Supplementary Note 1: Additional Algorithm Information

1.1 Feature preprocessing

We perform feature preprocessing to integrate both numerical covariates and categorical covariates. First for each categorical covariate, the categories are reordered based on the ratio of the alternative probability and the null probability, estimated on the training set using the same method as above. Then quantile normalization is performed for each covariate separately. Note that after this transformation, all covariates will have values between 0 and 1. Also, overfitting is not a concern since the entire proprecessing is done without seeing p-values from the testing set.

1.2 Remark on Theorem 1

Theorem 1 is similar to, but stronger than that for `NeuralFDR`. First, `NeuralFDR` requires the scale factor to be selected from a finite set of L numbers and has an extra multiplicative factor $\sqrt{\log L}$ in the error term ε. In contrast, `AdaFDR` selects the scale factor over all positive numbers and the $\sqrt{\log L}$ term is no longer needed. This is done by using a stochastic process argument instead of the union bound. Second, `NeuralFDR` uses an empirical Bayes model where the tuples $(P_i, \mathbf{x}_i, h_i)$ are generated i.i.d. following some hierarchical model. `AdaFDR`, however, requires a less restrictive assumption made only on the conditional distribution of null p-values, whereas the covariates and alternative p-values can have arbitrary dependence.

1.3 Initialization via EM algorithm

Here we present the EM algorithm that is used to fit the mixture model (2) on a set of N points $\{\mathbf{x}_i\}_{i=1}^N$. Recall that due to quantile normalization, the value of $\mathbf{x}_i$ is within $[0,1]^d$. Therefore, each component in the mixture model is truncated to be within $[0,1]^d$, i.e., truncated GLM or truncated Gaussian. Since we need to use the samples each associated with a sample weight, let us consider the general case where each sample $\mathbf{x}_i$ receives a positive weight $v_i \in \mathbb{R}_+$.

For the sake of convenience, let us reparameterize the parameters to have the standard probability distribution

$$f_{\text{all}}(\mathbf{x}; \mathbf{w}, \mathbf{a}, \{\boldsymbol{\mu}_k, \boldsymbol{\sigma}_k\}_{k=1}^K) = w_0 f_{\text{slope}}(\mathbf{x}; \mathbf{a}) + \sum_{k=1}^K w_k f_{\text{bump}}(\mathbf{x}; \boldsymbol{\mu}_k, \boldsymbol{\sigma}_k), \ \mathbf{x} \in [0,1]^d, \tag{1}$$

where $\mathbf{w} \in [0,1]^{K+1}$ with $\sum_{k=0}^K w_k = 1$ and

$$\begin{aligned} f_{\text{slope}}(\mathbf{x}; \mathbf{a}) &= \exp\left(\mathbf{a}^{\mathrm{T}}\mathbf{x}\right) \prod_{j=1}^d \frac{a_j}{\exp(a_j) - 1}, \\ f_{\text{bump}}(\mathbf{x}; \boldsymbol{\mu}_k, \boldsymbol{\sigma}_k) &= \prod_{j=1}^d \frac{1}{Z_{kj}\sqrt{2\pi\sigma_{kj}^2}} \exp\left(-\frac{(x_j - \mu_{kj})^2}{2\sigma_{kj}^2}\right), \\ \text{for } Z_{kj} &= \int_0^1 \frac{1}{\sqrt{2\pi\sigma_{kj}^2}} \exp\left(-\frac{(x_j - \mu_{kj})^2}{2\sigma_{kj}^2}\right) dx. \end{aligned}$$

It is not hard to see that (1) is equivalent to the mixture threshold (2) up to a scale factor that can be specified by b in (2); knowing one, the parameters for the other can be computed without difficulty.

The EM algorithm can be described as follows. For the initialization, the responsibility $\mathbf{r}_i \in [0,1]^{K+1}, i \in [N]$ for each point $\mathbf{x}_i$ is initialized as

$$\mathbf{r}_i^{\text{init}} = \left[0.5, \frac{1}{2K}, \frac{1}{2K}, \cdots, \frac{1}{2K}\right],$$

where the first component corresponds to the slope component and the rest correspond to the K bump components. Then, the algorithm iterates between the E-step and the M-step as follows until convergence:

1. **Expection (E-step)**: For each point $\mathbf{x}_i$, update the responsibility

$$\begin{aligned} \mathbf{r}_i^{\text{new}} = & \frac{1}{f_{\text{all}}(\mathbf{x}_i; \mathbf{w}^{\text{old}}, \mathbf{a}^{\text{old}}, \{\boldsymbol{\mu}_k^{\text{old}}, \boldsymbol{\sigma}_k^{\text{old}}\}_{k=1}^K)} [w_0^{\text{old}} f_{\text{slope}}(\mathbf{x}_i; \mathbf{a}^{\text{old}}), \\ & w_1^{\text{old}} f_{\text{bump}}(\mathbf{x}_i; \boldsymbol{\mu}_1^{\text{old}}, \boldsymbol{\sigma}_1^{\text{old}}), w_2^{\text{old}} f_{\text{bump}}(\mathbf{x}_i; \boldsymbol{\mu}_2^{\text{old}}, \boldsymbol{\sigma}_2^{\text{old}}), \cdots, w_K^{\text{old}} f_{\text{bump}}(\mathbf{x}_i; \boldsymbol{\mu}_K^{\text{old}}, \boldsymbol{\sigma}_K^{\text{old}})]. \end{aligned}$$

2. **Maximization (M-step)**: Update the component weights $\mathbf{w}^{\text{new}}$ by

$$w_k^{\text{new}} = \frac{\sum_{i=1}^N v_i w_{ik}^{\text{old}}}{\sum_{k=0}^K \sum_{i=1}^N v_i w_{ik}^{\text{old}}},\ k = 0, 1, \cdots, K$$

Update the parameters for the slope component and each of the K bump component:

$$\mathbf{a}^{\text{new}} = \text{ML}_{\text{slope}}(\{\mathbf{x}_i, v_i r_{i0}^{\text{new}}\}_{i=1}^N)$$
$$\boldsymbol{\mu}_k^{\text{new}}, \boldsymbol{\sigma}_k^{\text{new}} = \text{ML}_{\text{bump}}(\{\mathbf{x}_i, v_i r_{ik}^{\text{new}}\}_{i=1}^N),\ k \in [K].$$

The ML estimates of slope and bump, i.e., $\text{ML}_{\text{slope}}(\{\mathbf{x}_i, v_i r_{i0}^{\text{new}}\}_{i=1}^N)$ and $\text{ML}_{\text{bump}}(\{\mathbf{x}_i, v_i r_{ik}^{\text{new}}\}_{i=1}^N)$, are described as follows.
ML estimate of the slope. The log likelihood function of a single observation $\mathbf{x}_i$ can be written as

$$l_i(\mathbf{a}) = \log f_{\text{slope}}(\mathbf{x}_i; \mathbf{a}) = \sum_{j=1}^d \log\left(\frac{a_j}{\exp(a_j) - 1}\right) + \mathbf{a}^T \mathbf{x}_i. \tag{2}$$

Further the weighted average log likelihood function,

$$\bar{l}(\mathbf{a}) = \frac{\sum_{i=1}^N v_i r_{i0}^{\text{new}} l_i(\mathbf{a})}{\sum_{i=1}^N v_i r_{i0}^{\text{new}}} = \sum_{j=1}^d \log\left(\frac{a_j}{\exp(a_j) - 1}\right) + \frac{\mathbf{a}^T}{\sum_{i=1}^n v_i r_{i0}^{\text{new}}} \sum_{i=1}^N v_i r_{i0}^{\text{new}} \mathbf{x}_i. \tag{3}$$

We add a regularization term $c\|\mathbf{a}\|_2^2$ to encourage small values of $c\|\mathbf{a}\|_2^2$, i.e.

$$\bar{l}(\mathbf{a}) = \sum_{j=1}^d \log\left(\frac{a_j}{\exp(a_j) - 1}\right) + \frac{\mathbf{a}^T}{\sum_{i=1}^N v_i r_{i0}^{\text{new}}} \sum_{i=1}^N v_i r_{i0}^{\text{new}} \mathbf{x}_i - c\|\mathbf{a}\|_2^2. \tag{4}$$

We found that setting $c = 0.005$ gives a stable result. We solve the ML estimation problem by setting the derivative to be zero. Namely, for the jth element a_j,

$$\frac{\partial \bar{l}}{\partial a_j} = \frac{1}{a_j} - \frac{e^{a_j}}{e^{a_j} - 1} + \frac{1}{\sum_{i=1}^N v_i r_{i0}^{\text{new}}} \sum_{i=1}^N v_i r_{i0}^{\text{new}} x_{ij} - 2ca_j = 0. \tag{5}$$

Rearranging terms on both sides we have that the ML estimate $\hat{a}_j$ satisfies

$$\frac{e^{\hat{a}_j}}{e^{\hat{a}_j} - 1} - \frac{1}{\hat{a}_j} + 2c\hat{a}_j = \frac{1}{\sum_{i=1}^N v_i r_{i0}^{\text{new}}} \sum_{i=1}^N v_i r_{i0}^{\text{new}} x_{ij}. \tag{6}$$

Since the left-hand-side term is monotonic in $\hat{a}_j$, the ML solution $\hat{a}_j$ can be computed via binary search.
ML estimate of the k-th bump. Since the density function can be factorized as a product of different dimensions, the ML estimation can be done for each dimension separately. Now consider observation $\mathbf{x}_i$. The log likelihood function corresponding to dimension j can be written as

$$l_{ij}(\mu_{kj}, \sigma_{kj}) = -\log Z_{kj} - \frac{1}{2}\log(2\pi) - \log \sigma_{kj} - \frac{1}{2\sigma_{kj}^2}(x_{ij} - \mu_{kj})^2. \tag{7}$$

Then the weighted average log likelihood function for dimension j can be written as

$$\bar{l}_j(\mu_{kj}, \sigma_{kj}) = \frac{\sum_{i=1}^N v_i r_{ik}^{\text{new}} l_{ij}(\mu_{kj}, \sigma_{kj})}{\sum_{i=1}^N v_i r_{ik}^{\text{new}}} \tag{8}$$

$$= -\log Z_{kj} - \frac{1}{2}\log(2\pi) - \log \sigma_{kj} - \frac{1}{2\sigma_{kj}^2 \sum_{i=1}^n v_i r_{ik}^{\text{new}}} \sum_{i=1}^N v_i r_{ik}^{\text{new}} (x_{ij} - \mu_{kj})^2. \tag{9}$$

Since $\bar{l}_j(\mu_{kj}, \sigma_{kj})$ is convex, we compute the ML estimation $\hat{\mu}_{kj}$ and $\hat{\sigma}_{kj}$ via gradient descent, where the derivatives are given as follows.

$$\frac{\partial \bar{l}_j}{\partial \mu_{kj}} = -\frac{1}{Z_{kj}} \frac{\partial Z_{kj}}{\partial \mu_{kj}} + \frac{1}{\sigma_{kj}^2 \sum_{i=1}^N v_i r_{ik}^{\text{new}}} \sum_{i=1}^N v_i r_{ik}^{\text{new}} (x_{ij} - \mu_{kj}) \tag{10}$$

$$\frac{\partial \bar{l}_j}{\partial \sigma_{kj}} = -\frac{1}{Z_{kj}} \frac{\partial Z_{kj}}{\partial \sigma_{kj}} - \frac{1}{\sigma_{kj}} + \frac{1}{\sigma_{kj}^3 \sum_{i=1}^N v_i r_{ik}^{\text{new}}} \sum_{i=1}^N v_i r_{ik}^{\text{new}} (x_{ij} - \mu_{kj})^2, \tag{11}$$

where the derivatives with respect to Z_{kj} are

$$\frac{\partial Z_{kj}}{\partial \mu_{kj}} = \frac{1}{\sigma_{kj}}[\phi(\beta_1) - \phi(\beta_2)], \quad \frac{\partial Z_{kj}}{\partial \sigma_{kj}} = \frac{1}{\sigma_{kj}}[\beta_1\phi(\beta_1) - \beta_2\phi(\beta_2)], \tag{12}$$

for $\beta_1 = \frac{-\mu_{kj}}{\sigma_{kj}}$, $\beta_2 = \frac{1-\mu_{kj}}{\sigma_{kj}}$ and $\phi(x) = \frac{1}{\sqrt{2\pi}}\exp(-\frac{1}{2}x^2)$.

1.4 Extension to dependent case

Here we describe a simple procedure that extends `AdaFDR` to allow arbitrary dependency of p-values, borrowing ideas from the extended version of IHW[8]. The procedure can be described as follows:

1. Partition the hypotheses into two folds that are independent of each other, i.e., $\{(P_i, \mathbf{x}_i)\}_{i\in\mathscr{D}_1}$ and $\{(P_i, \mathbf{x}_i)\}_{i\in\mathscr{D}_2}$ that are mutually independent.

2. For each fold $j = 1,2$, let $\{t_i\}_{i\in\mathscr{D}_j}$ be the threshold learned from the other fold (up to a scaling factor). Weight the p-values by

$$\tilde{P}_i = P_i \frac{\sum_{i\in\mathscr{D}_j} t_i}{|\mathscr{D}_j| t_i},$$

   where $|\mathscr{D}_j|$ is the cardinality of the set $\mathscr{D}_j$.

3. Apply the BH procedure on the set of weighted p-values $\{\tilde{P}_i\}_{i\in[N]}$ with nominal FDR level $\alpha / \sum_{i\in[N]} \frac{1}{i}$.

We note that in eQTL studies, SNPs from different chromosomes can be regarded as being independent of each other. Also, the third step corresponds to the Benjamini-Yekutieli procedure[1]. By Theorem 1 in[8], the above procedure controls FDR under arbitrary dependency of p-values. More specifically, it controls FDR under the assumptions:

1. The two folds are independent of each other.

2. The null p-values, conditional on the covariates, are independent and stochastically greater than the uniform distribution.

As a side note, in practice, the dependent case will have minimum effect as long as the two folds are independent, i.e., step 1 in the procedure above.

1.5 Accelerate `AdaFDR`by data filtering using p-values

For input, `AdaFDR` also allows filtered data with only small p-values close to 0 and large p-values close to 1. This is because the mirror estimator only needs to look at these two parts of the data. In such case, the original number of hypotheses (before filtering) is required as input to control FDR (the argument `n_full` in the algorithm implementation). For the GTEx data, the data are filtered to have only data points with p-values $P_i < 0.01$ or $P_i > 0.99$, which greatly accelerated the algorithm. Let c_0 be the filtering threshold. Then for filtered data, only data points with p-values $P_i < c_0$ or $P_i > 1 - c_0$ are kept as input to `AdaFDR`. The filtering threshold c_0 should be much larger than the rejection threshold. For example, in the GTEx data, the rejection threshold can be smaller than 10^{-4} while the filtering threshold is chosen to be $c_0 = 0.01$.

1.6 Implementation of other methods

1. AdaPT: adapt_gam is used with a 5-degree spline for each dimension. This choice is based on a discussion with the authors of AdaPT.

2. IHW: The covaraites are first clustered into 20 clusters using Kmeans clustering. Then IHW is run with the default setting and the cluster label as the univariate covariate. This automatically incorporates the multi-dimensional case. For the univariate case, this does not change the result much as compared to directly running IHW. For example, for the airway data, directly running IHW gives 4873 discoveries while Kmeans+IHW gives 4862 discoveries.

3. BL: First the null distribution $\pi_0(\mathbf{x})$ is estimated using lm_pi0 with 5 degrees of freedom. Then BH is used with p-values weighted by $1/\pi_0(\mathbf{x}_i)$. This is the same as the usage in[11].

2 Supplementary Note 2: Data Information

Most data are available on GitHub repository (https://github.com/martinjzhang/AdaFDRpaper).

2.1 eQTL study

GTEx For eQTL study, we used Genotype-Tissue Expression (GTEx) dataset[4]. This dataset aims at characterizing variation in gene expression levels across individuals and diverse tissues of the human body. We used the V7 release of GTEx analysis data (dbGaP Accession phs000424.v7.p2). The dataset contains 11688 samples, and in total there are 53 tissues from 714 donors (44 of them with sample size >70 are used in the GTEx paper). We filtered the tissues based on the following criteria. First, the tissue needs to have eQTL analysis, where the number of samples with genotype is greater than 70. Second, we set the number of samples threshold to be 100 in order to make the p-values more reliable. Third, we would like the tissue to have a corresponding roadmap[2] cell type, so that we can leverage the cell-specific chromatin state data from roadmap. After filtering, we were left with 17 cell types. The meta-information of the filtered GTEx dataset is listed in Table 1.

Table 1. Information for selected GTEx tissue types

Tissue name	Sample size	Roadmap cell type	Number of hypothesis
Adipose Subcutaneous	298	E063	1.72E+08
Adipose Visceral Omentum	185	E063	1.73E+08
Artery Aorta	197	E065	1.66E+08
Breast Mammary Tissue	183	E027	1.80E+08
Cells EBV-transformed lymphocytes	114	E116	1.60E+08
Colon Sigmoid	124	E106	1.70E+08
Colon Transverse	169	E075, E076	1.77E+08
Esophagus Gastroesophageal Junction	127	E079	1.67E+08
Esophagus Mucosa	241	E079	1.67E+08
Esophagus Muscularis	218	E079	1.66E+08
Heart Atrial Appendage	159	E104	1.61E+08
Heart Left Ventricle	190	E095	1.50E+08
Lung	278	E096	1.82E+08
Muscle Skeletal	361	E107, E108	1.47E+08
Pancreas	149	E098	1.59E+08
Stomach	170	E110, E111	1.69E+08
Whole Blood	338	E062	1.45E+08

In this filtered dataset, each hypothesis is a gene-variant pair. Nominal P values for each gene-variant pair were estimated using a two-tailed t-test. Each gene-variant is associated with 4 or 5 covariates listed below:

- **gene expression** We obtained the median gene expression from the gene in gene-variant pair and used as a feature.
- **alternative allele frequency** We mapped each SNP to the dbSNP database[14]. We took the alternative allele frequency as a feature. If there were multiple alternative alleles, we took the smallest one. For the SNPs we cannot find a mapping, this feature is imputed with mean alternative allele frequency.
- **TSS distance** The distance from the SNP to the transcription starting site is used as a feature. It is defined as $pos_{SNP} - pos_{TSS}$.
- **cell-specific chromatin state** We took the position of the SNP and mapped it to roadmap database[2]. Each SNP falls into the 15-state chromatin model. This state is used as a categorical feature.
- **p-value from another tissue (optional)** Optionally, we used the P value from another tissue as a covariate. If we cannot find the same gene-variant pair in another tissue, we impute with the mean P value. This covariate is only used for "AdaFDR (aug)" and "AdaFDR (ctrl)" experiments.

Due to their large data size, we only provide the p-value filtered (< 0.01 or > 0.99) curated data for 17 all tissues in our online repository.

MuTHER In the Multiple Tissus Human Expression Resource project[5], samples from 850 individuals were collected and 3 tissues, namely adipose, LCL, and skin, were studied. We used only the data for the adipose tissue and the LCL tissue, where a nominal p-value is provided for each SNP-gene pair. Such curated MuTHER data is available in our online repository.

2.2 RNA-Seq data

We used three RNA-Seq datasets to validate our algorithm. The first one `bottomly`[3] is an RNA-Seq dataset used to detect differential gene expression between mouse strains. We used the same data preprocessing pipeline as in IHW[9]. p-values were calculated using `DESeq2`, and the mean of normalized counts for each gene were chosen to be the covariate. The second dataset `airway`[6] is an RNA-Seq dataset used to identify the differentially expressed genes in airway smooth muscle cell lines in response to dexamethasone. The dataset is processed with the same pipeline as `bottomly`. The thrid dataset `Pasilla`[7] is an RNA-Seq dataset for detecting genes that are differentially expressed between the normal and Pasilla-knockdown conditions. This dataset is available in `Pasilla` package and it is analyzed in the vignette of `genefilter` package using independent filtering method. The p-values were generated using `DESeq` package and the logarithm of normalized count were used as the covariate. All the preprocessing steps can be reproduced using vignettes of R package `IHWPaper`[10]. The data are available in our online repository.

2.3 Microbiome data

The two microbiome experiments are from the benchmark paper[11]. The data are available in our online repository.

2.4 Proteomics data

The proteomics data is from the IHW paper[9]. The data is available in our online repository.

2.5 fMRI data

The two fMRI data are from the fMRI paper[13]. The data are available in our online repository.

2.6 Simulated data

All simulated data generated (with different random seeds) are available in our online repository as data files.

Data 1. Simulated data with one covariate. The covariate $\mathbf{x}_i \sim \text{Unif}[0,1]$ and the probability of being an alternative hypothesis given the covariate $\mathbb{P}(h_i = 1|\mathbf{x}_i)$ is defined using the mixture model (1) as

$$\mathbb{P}(h_i = 1|\mathbf{x}_i) = 0.1 f_{\text{all}}(\mathbf{x}; \mathbf{w} = [0.5, 0.25, 0.25], a = 0.5, \mu_1 = 0.25, \mu_2 = 0.75, \sigma_1 = \sigma_2 = 0.05).$$

The null p-values are generated i.i.d. from Unif[0,1] while the alternative p-values are generated i.i.d. from $P_i \sim \text{Beta}(\alpha = 0.3, \beta = 4)$. The number of hypotheses is 20000 and 10 datasets are generated with different random seeds.

Data 2. Simulated data with two covariates The covariate $\mathbf{x}_i \sim \text{Unif}[0,1]$ and the probability of being an alternative hypothesis given the covariate $\mathbb{P}(h_i = 1|\mathbf{x}_i)$ is defined using the mixture model (1) as

$$\begin{aligned}\mathbb{P}(h_i = 1|\mathbf{x}_i) =& 0.1 f_{\text{all}}(\mathbf{x}; \mathbf{w} = [0.5, 0.25, 0.25], \mathbf{a} = [0.5, 0.5], \\ & \boldsymbol{\mu}_1 = [0.25, 0.25], \boldsymbol{\mu}_2 = [0.75, 0.75], \boldsymbol{\sigma}_1 = \boldsymbol{\sigma}_2 = [0.1, 0.1]).\end{aligned}$$

The null p-values are generated i.i.d. from Unif[0,1] while the alternative p-values are generated i.i.d. from $P_i \sim \text{Beta}(\alpha = 0.3, \beta = 4)$. The number of hypotheses is 20000 and 10 datasets are generated with different random seeds.

Data 3. Simulated data with ten covariates First a simulated data with two covariates is generated (data 2). Then, another 8 noisy dimensions are added to the covariates with each entry drawn i.i.d. from Unif[0,1]. The number of hypotheses is 20000 and 10 datasets are generated with different random seeds.

Data 4. Simulated data with weakly-dependent p-values The covariate $\mathbf{x}_i \sim \text{Unif}[0,1]$ and the probability of being an alternative hypothesis given the covariate $\mathbb{P}(h_i = 1|\mathbf{x}_i)$ is generated same as the simulated data with one covariate (data 1). The p-values are converted to z-scores via $p = 1 - \Phi(z)$, where $\Phi(\cdot)$ is the cdf of the standard normal distribution. Every 10 consecutive null z-scores are generated from $\mathcal{N}(0, \Sigma)$, while every 10 consecutive alternative z-scores are generated from $\mathcal{N}(2, \Sigma)$, with the symmetric covariance matrix whose upper triangular part is specified as

$$\begin{aligned}&\Sigma_{ii} = 1, \\ &\Sigma_{ij} = 0.25, i < j \leq 4, \\ &\Sigma_{ij} = -0.25, j > 4.\end{aligned}$$

We note instead of 0.25, the value 0.4 is used in the original paper (Section 3.2,[15]). However such choice makes the covariance matrix not positive semi-definite. We decrease the value until the matrix becomes positive semi-definite. The number of hypotheses is 20000 and 10 datasets are generated with different random seeds.

Data 5. Simulated data with strongly-dependent p-values The setting is the same as the weakly dependent data (data 4) except the generation of z-scores. Here, every 5 consecutive null z-scores are generated from $\mathcal{N}(0, I)$, while every 5 consecutive

alternative z-scores are generated from $\mathcal{N}(2, I)$. This perfect correlation means to model the linkage disequilibrium (LD) that frequently occurs in SNPs. Due to the reduction of the inherent multiplicity, the number of hypotheses is increased to 50000. 10 datasets are generated with different random seeds.

Data 6. Simulated data used in AdaPT The same data for Figure 6a in[12] is used where the number of hypotheses is 2500. 10 datasets are generated with different random seeds.

Data 7. Simulated data used in IHW The data is generated according to Supplementary Note 4.2.2 in[9] where the number of hypotheses is 20000. While the original paper varies the effect size from 1 to 2.5 (the shift of z-scores for alternative p-values), here we only use a fixed effect size 2. 10 datasets are generated with different random seeds.

3 Supplementary Note 3: Technical Proofs

3.1 Proof of Theorem 1

Proof. To avoid ambiguity, we make a few clarifications before the proof. First, the entire analysis is done while conditioning on the hypotheses splitting, all covariates $\{\mathbf{x}_i\}_{i\in[N]}$, the type of hypotheses $\{h_i\}_{i\in[N]}$, and the alternative p-values $\{P_i\}_{i\in\mathscr{H}_1}$, hence allowing arbitrary dependencies of them. Here we note that the reason for splitting the hypotheses at random is to attain good power. The randomness of the analysis comes from the null p-values, which are assumed to be i.i.d. uniformly distributed for convenience. A discussion on extending to the case where the null p-values, conditional on the covariates, are independently distributed and stochastically greater than the uniform distribution is provided at the end.

We also clarify a few notations. We use $t^*_{\mathscr{D}_1}$ to denote the threshold which is learned on fold 1 and will be applied on fold 2. γ^*_1 denotes the scale factor of fold 1. For the testing-related quantities, we use subscript "1" to denote those evaluated on fold 1, including the number of discoveries $\mathrm{D}_1(\gamma^*_1 t^*_{\mathscr{D}_2})$, the number of false discoveries $\mathrm{FD}_1(\gamma^*_1 t^*_{\mathscr{D}_2})$, the mirror-estimated number of false discoveries $\widehat{\mathrm{FD}}_1(\gamma^*_1 t^*_{\mathscr{D}_2})$ and the mirror-estimated false discovery proportion $\widehat{\mathrm{FDP}}_1(\gamma^*_1 t^*_{\mathscr{D}_2})$. Note that here $t^*_{\mathscr{D}_2}$ is the threshold that is learned on fold 2 and then applied on fold 1. The term inside the bracket, $(\gamma^*_1 t^*_{\mathscr{D}_2})$, may be omitted when there is no concern of being ambiguous. Quantities for fold 2 are defined in a similar fashion. Now we preceed to the proof.

Step 1: show that in order to prove the result, it suffices to show that

$$\mathbb{P}(\mathrm{FDP}_2 \geq (1+\varepsilon)\alpha) \leq \frac{\delta}{2}. \tag{13}$$

Indeed, if (13) it true, then by symmetry $\mathbb{P}(\mathrm{FDP}_1 \geq (1+\varepsilon)\alpha) \leq \frac{\delta}{2}$. Further by the union bound, with probability (w.p.) at least 1-δ,

$$\mathrm{FDP}_2 < (1+\varepsilon)\alpha, \text{ and } \mathrm{FDP}_1 < (1+\varepsilon)\alpha.$$

This further implies that w.p. at least 1-δ, the FDP on the whole dataset

$$\mathrm{FDP} = \frac{\mathrm{FD}_1 + \mathrm{FD}_2}{\mathrm{D}_1 + \mathrm{D}_2} \leq \left(\frac{\mathrm{FD}_1}{\mathrm{D}_1}\right) \vee \left(\frac{\mathrm{FD}_2}{\mathrm{D}_2}\right) = \mathrm{FDP}_1 \vee \mathrm{FDP}_2 < (1+\varepsilon)\alpha,$$

which gives the desired result. Hence in the rest of the proof, we denote effort to proving (13). Also, since we are only to deal with fold 2, we drop the subscript $\mathscr{D}_1$ for threshold learned on fold 1 to have $t^* \stackrel{def}{=} t^*_{\mathscr{D}_1}$.

Step 2: convert the probability $\mathbb{P}(\mathrm{FDP}_2 \geq (1+\varepsilon)\alpha)$ to some analyzable stochastic process.

Let $\mathscr{E}_0$ denote the set of random variables that we wish to condition on, including hypotheses splitting, all covariates $\{\mathbf{x}_i\}_{i\in[N]}$, the type of hypotheses $\{h_i\}_{i\in[N]}$, and the alternative p-values $\{P_i\}_{i\in\mathscr{H}_1}$. Let us consider the conditional version of (13):

$$\begin{aligned}\mathbb{P}(\mathrm{FDP}_2 \geq (1+\varepsilon)\alpha | \mathscr{E}_0) &= \mathbb{P}\left(\frac{\mathrm{FD}_2}{\mathrm{D}_2 \vee 1} \geq (1+\varepsilon)\alpha \middle| \mathscr{E}_0\right) \\ &= \mathbb{P}\left(\frac{\mathrm{FD}_2}{\mathrm{D}_2 \vee 1}\frac{1}{\alpha} - 1 \geq \varepsilon \middle| \mathscr{E}_0\right).\end{aligned}$$

Let $\eta \stackrel{def}{=} \left(\frac{\mathrm{FD}_2}{\mathrm{D}_2 \vee 1}\frac{1}{\alpha} - 1\right)$. Recall that FD_2 and D_2 correspond to the best rescaled threshold on fold 2 $\gamma^*_2 t^*$ and the best scale factor γ^*_2 is selected from the set $\left\{\gamma : \frac{\widehat{\mathrm{FD}}_2(\gamma t^*)}{\mathrm{D}_2(\gamma t^*)\vee 1} \leq \alpha, \mathrm{D}_2(\gamma t^*) \geq c_0 N\right\} \cup \{0\}$. Then η can be upper bounded by

$$\begin{aligned}\eta &= \frac{\mathrm{FD}_2(\gamma^*_2 t^*)}{\mathrm{D}_2(\gamma^*_2 t^*) \vee 1}\frac{1}{\alpha} - 1 \\ &\leq \sup_{\gamma \in \left\{\gamma:\ \frac{\widehat{\mathrm{FD}}_2(\gamma t^*)}{\mathrm{D}_2(\gamma t^*)\vee 1} \leq \alpha,\ \mathrm{D}_2(\gamma t^*) \geq c_0 N\right\} \cup \{0\}} \left(\frac{\mathrm{FD}_2(\gamma t^*)}{\mathrm{D}_2(\gamma t^*) \vee 1}\frac{1}{\alpha} - 1\right) \\ &\leq \sup_{\gamma \geq 0} \left(\frac{\mathrm{FD}_2(\gamma t^*)}{\mathrm{D}_2(\gamma t^*) \vee 1}\frac{1}{\alpha} - 1\right) \mathbb{I}_{\left\{\gamma:\ \frac{\widehat{\mathrm{FD}}_2(\gamma t^*)}{\mathrm{D}_2(\gamma t^*)\vee 1} \leq \alpha,\ \mathrm{D}_2(\gamma t^*) \geq c_0 N\right\}}.\end{aligned}$$

Furthermore, since the indicator function is one only when $\frac{\widehat{\mathrm{FD}}_2(\gamma t^*)}{\mathrm{D}_2(\gamma t^*)\vee 1} \leq \alpha$, which can also be written as $\frac{1}{\alpha} \leq \frac{\mathrm{D}_2(\gamma t^*)\vee 1}{\widehat{\mathrm{FD}}_2(\gamma t^*)}$ with the

convention that $\frac{x}{0} = \infty$ for any $x > 0$, we further have

$$\eta \leq \sup_{\gamma \geq 0} \left[\frac{\mathrm{FD}_2(\gamma t^*)}{\mathrm{D}_2(\gamma t^*) \vee 1} \left(\frac{1}{\alpha} \wedge \frac{\mathrm{D}_2(\gamma t^*) \vee 1}{\widehat{\mathrm{FD}}_2(\gamma t^*)} \right) - 1 \right] \mathbb{I}_{\left\{ \gamma:\ \frac{\widehat{\mathrm{FD}}_2(\gamma t^*)}{\mathrm{D}_2(\gamma t^*) \vee 1} \leq \alpha,\ \mathrm{D}_2(\gamma t^*) \geq c_0 N \right\}}$$
$$= \sup_{\gamma \geq 0} \left(\frac{\mathrm{FD}_2(\gamma t^*)}{(\alpha \mathrm{D}_2(\gamma t^*)) \vee \alpha \vee \widehat{\mathrm{FD}}_2(\gamma t^*)} - 1 \right) \mathbb{I}_{\left\{ \gamma:\ \frac{\widehat{\mathrm{FD}}_2(\gamma t^*)}{\mathrm{D}_2(\gamma t^*) \vee 1} \leq \alpha,\ \mathrm{D}_2(\gamma t^*) \geq c_0 N \right\}}.$$

Again since indicator function is one only when $\mathrm{D}_2(\gamma t^*) \geq c_0 N$,

$$\eta \leq \sup_{\gamma \geq 0} \left(\frac{\mathrm{FD}_2(\gamma t^*)}{(\alpha c_0 N) \vee \widehat{\mathrm{FD}}_2(\gamma t^*)} - 1 \right) \mathbb{I}_{\left\{ \gamma:\ \frac{\widehat{\mathrm{FD}}_2(\gamma t^*)}{\mathrm{D}_2(\gamma t^*) \vee 1} \leq \alpha,\ \mathrm{D}_2(\gamma t^*) \geq c_0 N \right\}},$$
$$\leq 0 \vee \sup_{\gamma \geq 0} \left(\frac{\mathrm{FD}_2(\gamma t^*)}{(\alpha c_0 N) \vee \widehat{\mathrm{FD}}_2(\gamma t^*)} - 1 \right).$$

Furthermore with the notation $t_i^* \stackrel{def}{=} t^*(\mathbf{x}_i)$ where we recall that we have defined $t^* = t^*_{\mathscr{D}_1}$ before,

$$\eta \leq 0 \vee \sup_{\gamma \geq 0} \left(\frac{\sum_{i \in \mathscr{D}_2 \cap \mathscr{H}_0} \mathbb{I}_{\{P_i \leq \gamma t_i^*\}}}{(\alpha c_0 N) \vee \left(\sum_{i \in \mathscr{D}_2} \mathbb{I}_{\{P_i \geq 1 - \gamma t_i^*\}} \right)} - 1 \right)$$
$$\leq 0 \vee \sup_{\gamma \geq 0} \left(\frac{\sum_{i \in \mathscr{D}_2 \cap \mathscr{H}_0} \mathbb{I}_{\{P_i \leq \gamma t_i^*\}}}{(\alpha c_0 N) \vee \left(\sum_{i \in \mathscr{D}_2 \cap \mathscr{H}_0} \mathbb{I}_{\{P_i \geq 1 - \gamma t_i^*\}} \right)} - 1 \right).$$

Finally, we can complete the conversion by noting that

$$\mathbb{P}(\mathrm{FDP}_2 \geq (1+\varepsilon)\alpha | \mathscr{E}_0) = \mathbb{P}(\eta \geq \varepsilon | \mathscr{E}_0) \tag{14}$$
$$\leq \mathbb{P}\left[\sup_{\gamma \geq 0} \left(\frac{\sum_{i \in \mathscr{D}_2 \cap \mathscr{H}_0} \mathbb{I}_{\{P_i \leq \gamma t_i^*\}}}{(\alpha c_0 N) \vee \left(\sum_{i \in \mathscr{D}_2 \cap \mathscr{H}_0} \mathbb{I}_{\{P_i \geq 1 - \gamma t_i^*\}} \right)} - 1 \right) \geq \varepsilon \middle| \mathscr{E}_0 \right]. \tag{15}$$

Here, the first term in (15), i.e. $\frac{\sum_{i \in \mathscr{D}_2 \cap \mathscr{H}_0} \mathbb{I}_{\{P_i \leq \gamma t_i^*\}}}{(\alpha c_0 N) \vee \left(\sum_{i \in \mathscr{D}_2 \cap \mathscr{H}_0} \mathbb{I}_{\{P_i \geq 1 - \gamma t_i^*\}} \right)}$, can be understood as a stochastic process that as γ grows from 0 to infinity, new elements are added to the numerator and the denominator with equal probability. Hence this term should always be close to 1. We next proceed to prove the result following this intuition.

Step 3: Upper bound the probability of (15).

We note that the p-values involved in (15) are all null p-values from fold 2. Hence, they are i.i.d. uniformly distributed conditional on $\mathscr{E}_0$. Let $\mathscr{H}_{0,2} \stackrel{def}{=} \mathscr{D}_2 \cap \mathscr{H}_0$. For any $i \in \mathscr{H}_{0,2}, \gamma > 0$, define the random variables

$$B_{i,\gamma} = \mathbb{I}_{\{P_i \leq \gamma t_i^* \ or \ P_i \geq 1 - \gamma t_i^*\}}, \quad R_i = \mathbb{I}_{\{P_i \leq 0.5\}} - \mathbb{I}_{\{P_i > 0.5\}}. \tag{16}$$

Since $\forall i \in \mathscr{H}_{0,2}$, $P_i | \mathscr{E}_0 \sim \mathrm{Unif}[0,1]$, we have $B_{i,\gamma} | \mathscr{E}_0 \sim \mathrm{Bern}(2\gamma t_i^*)$ and $R_i | \mathscr{E}_0$ are i.i.d. Rademacher random variables. In addition, it is easy to verify that $B_{i,\gamma}$ is independent of R_i and

$$\mathbb{I}_{\{P_i \leq \gamma t_i^*\}} = B_{i,\gamma} \mathbb{I}_{\{R_i = 1\}}, \quad \mathbb{I}_{\{P_i \geq 1 - \gamma t_i^*\}} = B_{i,\gamma} \mathbb{I}_{\{R_i = -1\}}.$$

Hence (15) can be written in terms of $B_{i,\gamma}$'s and R_i's as

$$\mathbb{P}(\mathrm{FDP}_2 \geq (1+\varepsilon)\alpha | \mathscr{E}_0) \leq \mathbb{P}\left[\sup_{\gamma \geq 0} \left(\frac{\sum_{i \in \mathscr{H}_{0,2}} B_{i,\gamma} \mathbb{I}_{\{R_i = 1\}}}{(\alpha c_0 N) \vee \sum_{i \in \mathscr{H}_{0,2}} B_{i,\gamma} \mathbb{I}_{\{R_i = -1\}}} - 1 \right) \geq \varepsilon \middle| \mathscr{E}_0 \right]$$
$$\leq \mathbb{P}\left[\sup_{\gamma \geq 0} \left(\frac{\sum_{i \in \mathscr{H}_{0,2}} B_{i,\gamma} R_i}{(\alpha c_0 N) \vee \sum_{i \in \mathscr{H}_{0,2}} B_{i,\gamma} \mathbb{I}_{\{R_i = -1\}}} \right) \geq \varepsilon \middle| \mathscr{E}_0 \right].$$

Furthermore, let $\gamma_0 = \frac{\alpha c_0 N}{\sum_{i\in\mathscr{H}_{0,2}} t_i^*}$. Divide the set of γ in the sup from $[0,\infty)$ into $[0,\gamma_0]$ and (γ_0,∞), and apply union bound to have

$$
\begin{aligned}
&\mathbb{P}(\mathrm{FDP}_2 \geq (1+\varepsilon)\alpha|\mathscr{E}_0)\\
&\leq \mathbb{P}\left[\sup_{0\leq\gamma\leq\gamma_0}\left(\frac{\sum_{i\in\mathscr{H}_{0,2}} B_{i,\gamma}R_i}{(\alpha c_0 N)\vee\sum_{i\in\mathscr{H}_{0,2}} B_{i,\gamma}\mathbb{I}_{\{R_i=-1\}}}\right)\geq\varepsilon\middle|\mathscr{E}_0\right]\\
&\quad+\mathbb{P}\left[\sup_{\gamma>\gamma_0}\left(\frac{\sum_{i\in\mathscr{H}_{0,2}} B_{i,\gamma}R_i}{(\alpha c_0 N)\vee\sum_{i\in\mathscr{H}_{0,2}} B_{i,\gamma}\mathbb{I}_{\{R_i=-1\}}}\right)\geq\varepsilon\middle|\mathscr{E}_0\right]\\
&\leq \mathbb{P}\left(\sup_{0\leq\gamma\leq\gamma_0}\frac{\sum_{i\in\mathscr{H}_{0,2}} B_{i,\gamma}R_i}{\alpha c_0 N}\geq\varepsilon\middle|\mathscr{E}_0\right)+\mathbb{P}\left(\sup_{\gamma>\gamma_0}\frac{\sum_{i\in\mathscr{H}_{0,2}} B_{i,\gamma}R_i}{\sum_{i\in\mathscr{H}_{0,2}} B_{i,\gamma}\mathbb{I}_{\{R_i=-1\}}}\geq\varepsilon\middle|\mathscr{E}_0\right).
\end{aligned}
$$

Define the random set $\mathscr{B}_\gamma = \{i : i\in\mathscr{H}_{0,2}, B_{i,\gamma}=1\}$. We note that the sequence of sets $\{\mathscr{B}_\gamma\}_{\gamma\geq 0}$ is monotonic in the sense that as γ grows, more elements are incorporated into $\mathscr{B}_\gamma$. With this definition, the above inequality can be further written as

$$
\begin{aligned}
&\mathbb{P}(\mathrm{FDP}_2 \geq (1+\varepsilon)\alpha|\mathscr{E}_0) &(17)\\
&\leq \mathbb{P}\left(\sup_{0\leq\gamma\leq\gamma_0}\frac{\sum_{i\in\mathscr{B}_\gamma} R_i}{\alpha c_0 N}\geq\varepsilon\middle|\mathscr{E}_0\right)+\mathbb{P}\left(\sup_{\gamma>\gamma_0}\frac{\sum_{i\in\mathscr{B}_\gamma} R_i}{\sum_{i\in\mathscr{B}_\gamma}\mathbb{I}_{\{R_i=-1\}}}\geq\varepsilon\middle|\mathscr{E}_0\right). &(18)
\end{aligned}
$$

Next we upper bound the two terms in (18) respectively. Here let us use $m \overset{def}{=} \alpha c_0 N$ for simplicity.
The first term in (18): For some $m_0 > 2m$ to be specified later, by the law of total probability,

$$
\begin{aligned}
&\text{first term in (18)} = \mathbb{P}\left(\sup_{0\leq\gamma\leq\gamma_0}\frac{\sum_{i\in\mathscr{B}_\gamma} R_i}{m}\geq\varepsilon\middle|\mathscr{E}_0\right) &(19)\\
&\leq \mathbb{P}\left(\sup_{0\leq\gamma\leq\gamma_0}\frac{\sum_{i\in\mathscr{B}_\gamma} R_i}{m}\geq\varepsilon, |\mathscr{B}_{\gamma_0}|\leq m_0\middle|\mathscr{E}_0\right)+\mathbb{P}\left(\sup_{0\leq\gamma\leq\gamma_0}\frac{\sum_{i\in\mathscr{B}_\gamma} R_i}{m}\geq\varepsilon, |\mathscr{B}_{\gamma_0}|> m_0\middle|\mathscr{E}_0\right) &(20)\\
&\leq \mathbb{P}\left(\sup_{0\leq\gamma\leq\gamma_0}\frac{\sum_{i\in\mathscr{B}_\gamma} R_i}{m}\geq\varepsilon\middle||\mathscr{B}_{\gamma_0}|\leq m_0,\mathscr{E}_0\right)+\mathbb{P}\left(|\mathscr{B}_{\gamma_0}|> m_0|\mathscr{E}_0\right). &(21)
\end{aligned}
$$

The two terms in (21) are upper bounded separately. Consider the first term. Recall that $\{\mathscr{B}_\gamma\}_{\gamma\geq 0}$ is a random sequence of monotonic sets; let $\{\tilde{\mathscr{B}}_\gamma\}_{\gamma\geq 0}$ denote any of its realization. Then since taking expectation over all possible $\{\tilde{\mathscr{B}}_\gamma\}_{\gamma\geq 0}$ s.t. $|\tilde{\mathscr{B}}_{\gamma_0}|\leq m_0$ is no greater than taking the sup of them,

$$
\text{first term in (21)} \leq \sup_{\{\tilde{\mathscr{B}}_\gamma\}_{\gamma\geq 0}\ s.t.\ |\tilde{\mathscr{B}}_{\gamma_0}|\leq m_0}\mathbb{P}\left(\sup_{0\leq\gamma\leq\gamma_0}\frac{\sum_{i\in\mathscr{B}_\gamma} R_i}{m}\geq\varepsilon\middle|\{\mathscr{B}_\gamma\}_{\gamma\geq 0}=\{\tilde{\mathscr{B}}_\gamma\}_{\gamma\geq 0},\mathscr{E}_0\right).
$$

Consider the term inside the probability, i.e. $\sup_{0\leq\gamma\leq\gamma_0}\frac{\sum_{i\in\mathscr{B}_\gamma} R_i}{m}$, where due to conditioning $\{\mathscr{B}_\gamma\}_{\gamma\geq 0}=\{\tilde{\mathscr{B}}_\gamma\}_{\gamma\geq 0}$. Recall that the sequence $\{\tilde{\mathscr{B}}_\gamma\}_{\gamma\geq 0}$ is monotonic that as γ grows more elements are incorporated into the set but no element is removed from the set. Also up to the point $\gamma=\gamma_0$ there are altogether $|\tilde{\mathscr{B}}_{\gamma_0}|$ elements. Then the sup is equivalent to being evaluated over a sequence of $|\tilde{\mathscr{B}}_{\gamma_0}|+1$ monotonic sets, i.e. $\sup_{0\leq\gamma\leq\gamma_0}\frac{\sum_{i\in\mathscr{B}_\gamma} R_i}{m}$ is equal to $\sup_{0\leq k\leq|\tilde{\mathscr{B}}_{\gamma_0}|}\frac{\sum_{i\in[k]}\tilde{R}_i}{m}$ in distribution, where $\tilde{R}_1,\tilde{R}_2,\cdots$ is a sequence of i.i.d. Rademacher random variables independent of everything else. Therefore,

$$
\begin{aligned}
\text{first term in (21)} &\leq \sup_{\{\tilde{\mathscr{B}}_\gamma\}_{\gamma\geq 0}\ s.t.\ |\tilde{\mathscr{B}}_{\gamma_0}|\leq m_0}\mathbb{P}\left(\max_{0\leq k\leq|\tilde{\mathscr{B}}_{\gamma_0}|}\frac{\sum_{i\in[k]}\tilde{R}_i}{m}\geq\varepsilon\right)\\
&= \mathbb{P}\left(\max_{1\leq k\leq m_0}\frac{\sum_{i\in[k]}\tilde{R}_i}{m}\geq\varepsilon\right)\leq 2e^{-\frac{m^2\varepsilon^2}{2m_0}},
\end{aligned}
$$

where the last inequality is due to Lemma 1.

Now consider the second term in (21). Recall that $\mathbb{E}[|\mathscr{B}_{\gamma_0}|] = \sum_{i\in\mathscr{H}_{0,2}} 2\gamma_0 t_i = 2m$ by the definition of γ_0. By Lemma 2,

$$\text{second term in (21)} = \mathbb{P}\left[|\mathscr{B}_{\gamma_0}| > m_0|\mathscr{E}_0\right] \le e^{-\frac{\frac{1}{2}(m_0-2m)^2}{2m+\frac{1}{3}(m_0-2m)}}. \tag{22}$$

By setting $m_0 = 3m$, we have

$$\text{first term in (18)} = \mathbb{P}\left(\sup_{0\le\gamma\le\gamma_0} \frac{\sum_{i\in\mathscr{B}_\gamma} R_i}{m} \ge \varepsilon \middle| \mathscr{E}_0\right) \le 2e^{-\frac{m\varepsilon^2}{6}} + e^{-\frac{3m}{14}}. \tag{23}$$

The second term in (18): For some $m_1 \le 2m$ to be specified later, by the law of total probability,

$$\text{second term in (18)} = \mathbb{P}\left(\sup_{\gamma>\gamma_0} \frac{\sum_{i\in\mathscr{B}_\gamma} R_i}{\sum_{i\in\mathscr{B}_\gamma} \mathbb{I}_{\{R_i=-1\}}} \ge \varepsilon \middle| \mathscr{E}_0\right) \tag{24}$$

$$= \mathbb{P}\left(\sup_{\gamma>\gamma_0} \frac{\sum_{i\in\mathscr{B}_\gamma} R_i}{\sum_{i\in\mathscr{B}_\gamma} \mathbb{I}_{\{R_i=-1\}}} \ge \varepsilon, |\mathscr{B}_{\gamma_0}| \ge m_1 \middle| \mathscr{E}_0\right) + \mathbb{P}\left(\sup_{\gamma>\gamma_0} \frac{\sum_{i\in\mathscr{B}_\gamma} R_i}{\sum_{i\in\mathscr{B}_\gamma} \mathbb{I}_{\{R_i=-1\}}} \ge \varepsilon, |\mathscr{B}_{\gamma_0}| < m_1 \middle| \mathscr{E}_0\right) \tag{25}$$

$$\le \mathbb{P}\left(\sup_{\gamma>\gamma_0} \frac{\sum_{i\in\mathscr{B}_\gamma} R_i}{\sum_{i\in\mathscr{B}_\gamma} \mathbb{I}_{\{R_i=-1\}}} \ge \varepsilon \middle| |\mathscr{B}_{\gamma_0}| \ge m_1, \mathscr{E}_0\right) + \mathbb{P}\left(|\mathscr{B}_{\gamma_0}| < m_1|\mathscr{E}_0\right). \tag{26}$$

Using the same argument for analyzing the first term in (21),

$$\text{first term in (26)} \le \mathbb{P}\left(\sup_{k\ge m_1} \frac{\sum_{i\in[k]} \tilde{R}_i}{\sum_{i\in[k]} \mathbb{I}_{\{\tilde{R}_i=-1\}}} \ge \varepsilon\right) \le \frac{2e^{-\frac{m_1\varepsilon^2}{4(\varepsilon+2)^2}}}{1-2e^{-\frac{m_1\varepsilon^2}{4(\varepsilon+2)^2}}},$$

where we recall that $\tilde{R}_1, \tilde{R}_2, \cdots$ is a sequence of i.i.d. Rademacher random variables indepedent of everything else, and the second inequality is due to Lemma 1.

Similar to (22), by Lemma 2,

$$\text{second term in (26)} = \mathbb{P}\left(|\mathscr{B}_{\gamma_0}| < m_1\right) \le e^{-\frac{\frac{1}{2}(2m-m_1)^2}{2m+\frac{1}{3}(2m-m_1)}}.$$

By setting $m_1 = m$, we have

$$\text{second term in (18)} = \mathbb{P}\left(\sup_{\gamma>\gamma_0} \frac{\sum_{i\in\mathscr{B}_\gamma} R_i}{\sum_{i\in\mathscr{B}_\gamma} \mathbb{I}_{\{R_i=-1\}}} \ge \varepsilon \middle| \mathscr{E}_0\right) \le \frac{2e^{-\frac{m\varepsilon^2}{4(\varepsilon+2)^2}}}{1-2e^{-\frac{m\varepsilon^2}{4(\varepsilon+2)^2}}} + e^{-\frac{3m}{14}}. \tag{27}$$

Combining (23) and (27) we have that for (18),

$$\mathbb{P}(\text{FDP}_2 \ge (1+\varepsilon)\alpha|\mathscr{E}_0) \le 2e^{-\frac{m\varepsilon^2}{6}} + \frac{2e^{-\frac{m\varepsilon^2}{4(\varepsilon+2)^2}}}{1-2e^{-\frac{m\varepsilon^2}{4(\varepsilon+2)^2}}} + 2e^{-\frac{3m}{14}}.$$

Furthermore,

$$\mathbb{P}(\text{FDP}_2 \ge (1+\varepsilon)\alpha) \le \sup_{\mathscr{E}_0} \mathbb{P}(\text{FDP}_2 \ge (1+\varepsilon)\alpha|\mathscr{E}_0) \tag{28}$$

$$\le 2e^{-\frac{m\varepsilon^2}{6}} + + \frac{2e^{-\frac{m\varepsilon^2}{4(\varepsilon+2)^2}}}{1-2e^{-\frac{m\varepsilon^2}{4(\varepsilon+2)^2}}} + 2e^{-\frac{3m}{14}}. \tag{29}$$

By equaling the term in the right-hand-side of (29) with $\frac{\delta}{2}$ we have $\varepsilon = \Theta(\sqrt{\frac{\log\frac{1}{\delta}}{m}})$. Recall that $m = \alpha c_0 N$ where c_0 is a constant, we have

$$\varepsilon = \Theta(\sqrt{\frac{\log\frac{1}{\delta}}{\alpha N}}),$$

which concludes the proof.

In order for the proof to hold, it is required that the mirror estimate $\widehat{\mathrm{FD}}_2(\gamma t^*)$ is stochastically no less than the true number of false discoveries $\mathrm{FD}_2(\gamma t^*)$ for any $\gamma \geq 0$. This is still true when the i.i.d. assumption for the null p-values is extended to the assumption that the null p-values, conditional on the covariates, are independently distributed and stochastically greater than the uniform distribution. Hence the result is directly extendable. □

3.2 Lemma 1 with proof

Lemma 1. *(Some properties of random walk) Let $R_1, R_2, \cdots$ be i.i.d. Rademacher random variables and let $S_k = \sum_{i=1}^k R_k$. Then for any integer $n > 1$ and for any real number $t > 0$,*

$$\mathbb{P}(\max_{1\leq k\leq n} S_k \geq t) \leq 2e^{-\frac{t^2}{2n}} \tag{30}$$

$$\mathbb{P}(\max_{k\geq n} \frac{1}{k} S_k \geq t) \leq \frac{2e^{-\frac{nt^2}{4}}}{1-2e^{-\frac{nt^2}{4}}} \tag{31}$$

$$\mathbb{P}(\max_{k\geq n} \frac{S_k}{\sum_{i=1}^k \mathbb{I}_{\{R_i=-1\}}} \geq t) \leq \frac{2e^{-\frac{nt^2}{4(t+2)^2}}}{1-2e^{-\frac{nt^2}{4(t+2)^2}}}, \tag{32}$$

where for the second and the third inequalities, we require t to be large enough for the probability to be positive.

Proof. (30) is proved via a standard reflection argument for random walk. First consider when t is an integer,

$$\begin{aligned}
&\mathbb{P}(\max_{1\leq k\leq n} S_k \geq t) = \mathbb{P}(\max_{1\leq k\leq n} S_k \geq t, S_n \geq t) + \mathbb{P}(\max_{1\leq k\leq n} S_k \geq t, S_n < t) \\
&= \mathbb{P}(S_n \geq t) + \mathbb{P}(\max_{1\leq k\leq n} S_k \geq t, S_n > t) = \mathbb{P}(S_n \geq t) + \mathbb{P}(S_n > t) \leq 2\mathbb{P}(S_n \geq t).
\end{aligned}$$

If t is not an integer,

$$\mathbb{P}(\max_{1\leq k\leq n} S_k \geq t) = \mathbb{P}(\max_{1\leq k\leq n} S_k \geq \lceil t \rceil) \leq 2\mathbb{P}(S_n \geq \lceil t \rceil) \leq 2\mathbb{P}(S_n \geq t).$$

Finally, using Hoeffding's inequality, one has

$$\mathbb{P}(\max_{1\leq k\leq n} S_k \geq t) \leq 2\mathbb{P}(S_n \geq t) \leq 2e^{-\frac{t^2}{2n}}.$$

(31) is proved via a technique called "peeling". Specifically,

$$\begin{aligned}
&\mathbb{P}(\max_{k\geq n} \frac{1}{k} S_k \geq t) \leq \mathbb{P}(\exists k \geq n, S_k \geq kt) \\
&\leq \sum_{j=0}^{\infty} \mathbb{P}(\exists k \in \{2^j n, 2^j n+1, \cdots, 2^{j+1}n-1\}, S_k \geq kt) \\
&\leq \sum_{j=0}^{\infty} \mathbb{P}(\exists k \in \{2^j n, 2^j n+1, \cdots, 2^{j+1}n-1\}, S_k \geq 2^j nt) \\
&\leq \sum_{j=0}^{\infty} \mathbb{P}(\max_{1\leq k\leq 2^{j+1}n} S_k \geq 2^j nt) \\
&\leq \sum_{j=0}^{\infty} 2\exp(-2^{j-2}nt^2) \overset{def}{=} \sum_{j=0}^{\infty} p_j,
\end{aligned}$$

where the last inequality is due to (30) that we have just proved. Note that for $j \geq 0$, $\frac{p_{j+1}}{p_j} = \exp(-2^{j-2}nt^2) \leq p_0$. Hence,

$$\mathbb{P}(\max_{k\geq n} \frac{1}{k} S_k \geq t) \leq \frac{p_0}{1-p_0} = \frac{2e^{-\frac{nt^2}{4}}}{1-2e^{-\frac{nt^2}{4}}}.$$

Finally, (32) is a direct consequence of (31):

$$\mathbb{P}(\max_{k\geq n}\frac{S_k}{\sum_{i=1}^{k}\mathbb{I}_{\{R_i=-1\}}}\geq t)=\mathbb{P}(\max_{k\geq n}\frac{2S_k}{k-S_k}\geq t)$$

$$=\mathbb{P}(\max_{k\geq n}\frac{1}{k}S_k\geq\frac{t}{t+2})\leq\frac{2e^{-\frac{nt^2}{4(t+2)^2}}}{1-2e^{-\frac{nt^2}{4(t+2)^2}}}.$$

□

3.3 Lemma 2 with proof

Lemma 2. *(Some properties of non-homogeneous Bernoulli sum) Let $B_i\sim Bern(p_i)$ be some independent Bernoulli random variables. Then*

$$\mathbb{P}(\sum_{i=1}^{n}B_i-\mathbb{E}[\sum_{i=1}^{n}B_i]\geq t)\leq e^{-\frac{\frac{1}{2}t^2}{\sum_{i=1}^{n}p_i+\frac{1}{3}t}} \tag{33}$$

$$\mathbb{P}(\sum_{i=1}^{n}B_i-\mathbb{E}[\sum_{i=1}^{n}B_i]\leq -t)\leq e^{-\frac{\frac{1}{2}t^2}{\sum_{i=1}^{n}p_i+\frac{1}{3}t}} \tag{34}$$

Proof. Define $X_i\overset{def}{=}B_i-p_i$. Then X_i's have zero means and are independent of each other. Also, note that $|X_i|\leq 1$ almost surely and $\sum_i\mathbb{E}[X_i^2]\leq\sum_i p_i$. Hence (33) and (34) can be obtained by applying Bernstein inequality on $\{X_i\}$ and $\{-X_i\}$ respectively.

□

References

1. Yoav Benjamini and Daniel Yekutieli. The control of the false discovery rate in multiple testing under dependency. *Annals of statistics*, pages 1165–1188, 2001.
2. Bradley E Bernstein, John A Stamatoyannopoulos, Joseph F Costello, Bing Ren, Aleksandar Milosavljevic, Alexander Meissner, Manolis Kellis, Marco A Marra, Arthur L Beaudet, Joseph R Ecker, et al. The nih roadmap epigenomics mapping consortium. *Nature biotechnology*, 28(10):1045, 2010.
3. Daniel Bottomly, Nicole AR Walter, Jessica Ezzell Hunter, Priscila Darakjian, Sunita Kawane, Kari J Buck, Robert P Searles, Michael Mooney, Shannon K McWeeney, and Robert Hitzemann. Evaluating gene expression in c57bl/6j and dba/2j mouse striatum using rna-seq and microarrays. *PloS one*, 6(3):e17820, 2011.
4. GTEx Consortium et al. Genetic effects on gene expression across human tissues. *Nature*, 550(7675):204, 2017.
5. Elin Grundberg, Kerrin S Small, Åsa K Hedman, Alexandra C Nica, Alfonso Buil, Sarah Keildson, Jordana T Bell, Tsun-Po Yang, Eshwar Meduri, Amy Barrett, et al. Mapping cis-and trans-regulatory effects across multiple tissues in twins. *Nature genetics*, 44(10):1084, 2012.
6. Blanca E Himes, Xiaofeng Jiang, Peter Wagner, Ruoxi Hu, Qiyu Wang, Barbara Klanderman, Reid M Whitaker, Qingling Duan, Jessica Lasky-Su, Christina Nikolos, et al. Rna-seq transcriptome profiling identifies crispld2 as a glucocorticoid responsive gene that modulates cytokine function in airway smooth muscle cells. *PloS one*, 9(6):e99625, 2014.
7. Wolfgang Huber. Reyes, a. pasilla: Data package with per-exon and per-gene read counts of rna-seq samples of pasilla knock-down by brooks et al. *Genome Research*, 2011.
8. Nikolaos Ignatiadis and Wolfgang Huber. Covariate-powered weighted multiple testing with false discovery rate control. *Preprint at https://arxiv.org/abs/1701.05179*, 2017.
9. Nikolaos Ignatiadis, Bernd Klaus, Judith B Zaugg, and Wolfgang Huber. Data-driven hypothesis weighting increases detection power in genome-scale multiple testing. *Nature methods*, 13(7):577–580, 2016.
10. Nikos Ignatiadis. *IHWpaper: Reproduce figures in IHW paper*, 2018. R package version 1.7.0.
11. Keegan Korthauer, Patrick K Kimes, Claire Duvallet, Alejandro Reyes, Ayshwarya Subramanian, Mingxiang Teng, Chinmay Shukla, Eric J Alm, and Stephanie C Hicks. A practical guide to methods controlling false discoveries in computational biology. *Preprint at https://www.biorxiv.org/content/10.1101/458786v1*, 2018.
12. Lihua Lei and William Fithian. Adapt: an interactive procedure for multiple testing with side information. *Journal of the Royal Statistical Society: Series B (Statistical Methodology)*, 80(4):649–679, 2018.
13. Konstantin Schildknecht, Karsten Tabelow, and Thorsten Dickhaus. More specific signal detection in functional magnetic resonance imaging by false discovery rate control for hierarchically structured systems of hypotheses. *PloS one*, 11(2):e0149016, 2016.
14. Stephen T Sherry, M-H Ward, M Kholodov, J Baker, Lon Phan, Elizabeth M Smigielski, and Karl Sirotkin. dbsnp: the ncbi database of genetic variation. *Nucleic acids research*, 29(1):308–311, 2001.
15. John D Storey, Jonathan E Taylor, and David Siegmund. Strong control, conservative point estimation and simultaneous conservative consistency of false discovery rates: a unified approach. *Journal of the Royal Statistical Society: Series B (Statistical Methodology)*, 66(1):187–205, 2004.
